# Complete chloroplast genome sequences of *Lilium*: insights into evolutionary dynamics and phylogenetic analyses

**DOI:** 10.1038/s41598-017-06210-2

**Published:** 2017-07-18

**Authors:** Yun-peng Du, Yu Bi, Feng-ping Yang, Ming-fang Zhang, Xu-qing Chen, Jing Xue, Xiu-hai Zhang

**Affiliations:** 1 Beijing Agro-Biotechnology Research Center, Beijing Key Laboratory of Agricultural Genetic Resources and Biotechnology; Key Laboratory of Urban Agriculture (North), Ministry of Agriculture, Beijing Academy of Agriculture and Forestry Sciences, Beijing, 100097 China; 2 School of Horticulture, Jilin Agricultural University, Changchun, Jilin Province 130000 China

## Abstract

*Lilium* is a large genus that includes approximately 110 species distributed throughout cold and temperate regions of the Northern Hemisphere. The species-level phylogeny of *Lilium* remains unclear; previous studies have found universal markers but insufficient phylogenetic signals. In this study, we present the use of complete chloroplast genomes to explore the phylogeny of this genus. We sequenced nine *Lilium* chloroplast genomes and retrieved seven published chloroplast genomes for comparative and phylogenetic analyses. The genomes ranged from 151,655 bp to 153,235 bp in length and had a typical quadripartite structure with a conserved genome arrangement and moderate divergence. A comparison of sixteen *Lilium* chloroplast genomes revealed ten mutation hotspots. Single nucleotide polymorphisms (SNPs) for any two *Lilium* chloroplast genomes ranged from 8 to 1,178 and provided robust data for phylogeny. Except for some of the shortest internodes, phylogenetic relationships of the *Lilium* species inferred from the chloroplast genome obtained high support, indicating that chloroplast genome data will be useful to help resolve the deeper branches of phylogeny.

## Introduction

The genus *Lilium*, in the family Liliaceae, is economically and phylogenetically important and includes approximately 110 species distributed throughout the cold and temperate regions of the Northern Hemisphere, especially East Asia and North America^1, 2^, with eastern Asia and the Himalayas established as the center of diversity for this species. De Jong^3^ and Patterson & Givnish^4^ have described southwestern China and the Himalayas as the point of origin for the genus *Lilium*. Many *Lilium* species, ornamental cultivars and hybrids (such as Oriental hybrid, LA-hybrid, OT-hybrid, Asiatic hybrid, LO-hybrid, Longiflorum, and Aurelian & Trumpet), are cultivated for their esthetic value. In addition, both the flowers and bulbs are regularly consumed as both food and medicine in many parts of the world, particularly in Asia^5^. Presently, the “medicine food homology” values of *Lilium* plants have received considerable attention with respect to their great commercial prospects.

Nevertheless, many natural distribution areas of the wild lily are being adversely affected by both natural and human forces^6, 7^, and a growing number of *Lilium* species are on the verge of extinction (the IUCN Red List of Threatened Species (http://www.iucnredlist.org)). Thus, programs to protect and preserve lily resources (especially rare lily species) are urgently needed. Species endemic to China—*L. paradoxum* Stearn, *L. medogense* S. Y. Liang, *L. pinifolium* L. J. Peng, *L. saccatum* S. Y. Liang, *L. huidongense* J. M. Xu, *L. matangense* J. M. Xu, *L. stewartianum* I. B. Balfour et W. W. Smith, *L. habaense* F. T. Wang et Tang, *L. jinfushanense* L. J. Peng et B. N. Wang, *L. xanthellum* F. T. Wang et Tang and *L. fargesii* Franch.—have been put on the China Species Red List^8^.

*Lilium*, which is taxonomically and phylogenetically regarded as an important clade of the core Liliales, appears to have evolved in the Himalayas approximately 12 million years ago, despite the lack of fossil records^4, 9^. Currently, the major phylogenetic clades of *Lilium* have been basically clear, and the updated system classifies the genus into seven sections primarily based on morphological taxonomy and molecular phylogenetic methods^10–15^. In the past two decades, the nuclear rDNA internal transcribed spacer (ITS)^11, 13–15^ and several plastid genome regions have frequently been used in *Lilium* molecular systematics, including *matK*, *rbcL*, *ndhF*, and spacer regions of *trnL-F*, *rpl32-trnL*, *trnH-psbA*, or their combination. In addition, these studies have shown an incongruence between plastid and nuclear phylogenies^4, 12, 16^. A similar incongruence has been reported in recent studies of the genus *Oryza* ^17^, the tribe Arundinarieae^18^, the genera *Medicago* ^19^ and *Ilex* ^20^ and has been attributed to the use of markers with insufficient phylogenetic signals, incomplete lineage sorting, or complex evolutionary issues. Moreover, the selected loci unfortunately have not provided sufficient phylogenetic resolution at the species level for *Lilium*. For the conservation, utilization, and domestication of *Lilium* plants, more effective molecular markers are needed to identify *Lilium* species and evaluate the population genetics and breeding for the *Lilium* genus. DNA barcoding can be used to elucidate plant relationships at the species level; therefore, the identification of high-resolution molecular markers at the species level is critical to the success of DNA barcoding in plants^21^.

Chloroplast (cp) is the key organelle for photosynthesis and carbon fixation in green plants^22^, and therefore, their genomes could provide valuable information for taxonomic classification and the reconstruction of phylogeny because of sequence divergence among plant species and individuals^23^. Due to their maternal inheritance, very low recombination and haploidy, cp genomes are helpful for tracing source populations and phylogenetic studies of land plants for resolving complex evolutionary relationships^24–26^. Typical cp genomes in angiosperms have a generally conserved quadripartite circular structure with two copies of inverted repeat (IR) regions that are separated by a large single copy (LSC) region and a small single copy (SSC) region^27, 28^. These genomes with sizes in the range of 120–170 kb typically encode 120–130 genes.

The use of whole chloroplast genomes as a universal barcode and the existence of variable characters among the chloroplast genomes at the species level have recently been demonstrated, helping to overcome the previously low resolution in plant relationships^17, 20–23, 29–33^. With the rapid development of next-generation sequencing, it is now more convenient and relatively inexpensive to obtain cp genome sequences and extend gene-based phylogenetics to phylogenomics.

In this study, we present the complete chloroplast genomes of nine *Lilium* species through NGS sequencing and add seven species from GenBank^34–40^. We then test the feasibility of phylogeny reconstruction using the chloroplast genome. We further perform an analysis to gain insights into the overall evolutionary dynamics of chloroplast genomes in *Lilium*.

## Results

### Genome sequencing and assembly

Using the Illumina HiSeq 4000 system, nine *Lilium* taxa were sequenced to produce 3,719,304–11,167,835 paired-end raw reads (150 bp in average read length). *Lilium* cp genomes were *de novo* assembled using SPAdes 3.6.1. After these paired-end reads were screened through alignment with the chloroplast genome using Geneious V9, 47,505 to 988,478 cp genome reads were extracted with 46 X to 971 X coverage (Table 1). The four junction regions in each genome were validated by PCR-based sequencing according to Dong *et al*.^41^.

**Table 1:** Summary of the sequencing data for nine *Lilium* species.

Species	Raw data no.	Mapped read no.	Mapped to reference genome (%)	cp gemome coverage (X)
*L. fargesii*	3719304	47505	0.64%	46.50
*L. brownii*	8014171	988478	6.17%	971.15
*L. lancifolium*	5702538	174796	1.53%	171.85
*L. nepalense* var. *ochraceum*	6346201	248794	1.96%	245.03
*L. leucanthum*	5962131	131349	1.10%	128.83
*L. davidii* var. *willmottiae*	7409181	369289	2.49%	362.86
*L. duchartrei*	11167835	185719	0.83%	182.93
*L. bakerianum*	6897215	74085	0.54%	73.28
*L. henryi*	9331411	322449	1.73%	315.88

### Complete chloroplast genomes of *Lilium* species

**Figure 1:**
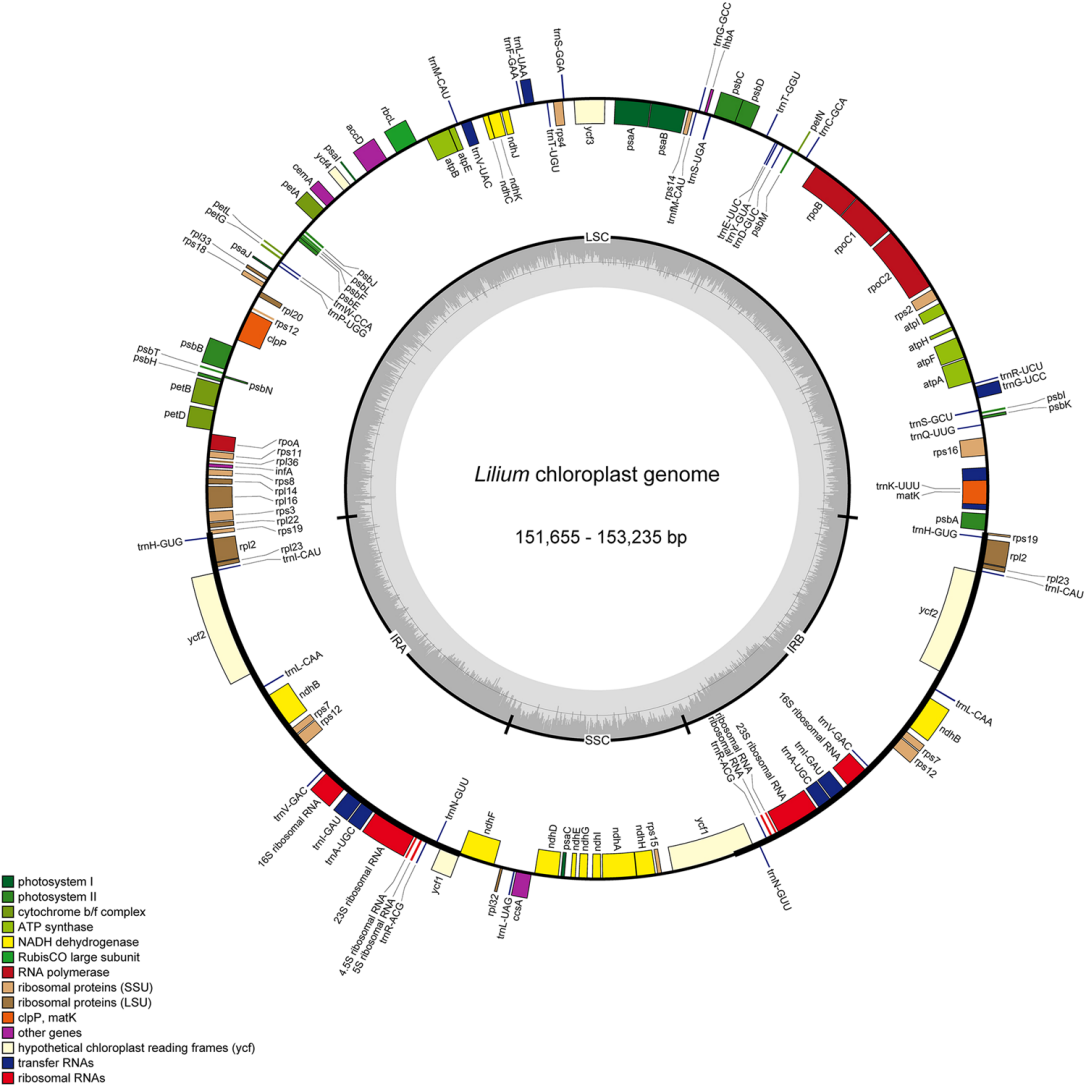
Gene map of the 16 *Lilium* chloroplast genome. The genes inside and outside of the circle are transcribed in the clockwise and counterclockwise directions, respectively. Genes belonging to different functional groups are shown in different colors. The thick lines indicate the extent of the inverted repeats (IRa and IRb) that separate the genomes into small single copy (SSC) and large single copy (LSC) regions.

The nucleotide sequences of the 16 *Lilium* cp genomes range from 151,655 bp (*L. bakerianum*) to 153,235 bp (*L. fargesii*; Fig. 1, Table 2). The Chloroplast genomes assembled in single circular, double-stranded DNA sequences, displaying a typical quadripartite structure, consisting of a pair of IRs (26,394–26,990 bp) separated by the LSC (81,224–82,480 bp) and SSC (17,038–17,620 bp) regions. The overall GC content is 36.9–37.1%, indicating nearly identical levels among the 16 complete *Lilium* cp genomes. The *Lilium* cp genome contains 113 genes, including 79 protein coding genes, 30 tRNA genes, and 4 rRNA genes (Fig. 1, Table S1). All four rRNA genes are duplicated in the IR region. Fifteen distinct genes contain one intron, two of which contain two introns (*clpP* and *ycf3*). The *rps12* gene is a trans-spliced gene with the 5′ end located in the LSC region and the duplicated 3′ end in the IR region, as has been reported previously in other plants.

**Table 2:** Summary of complete chloroplast genomes of *Lilium* species.

Species	Total	LSC	IR	SSC	Total	Protein coding genes	tRNA	rRNA	GC%	Accession number in Genbank
*L. bakerianum*	151655	81224	26423	17585	113	79	30	4	37.1	KY748301
*L. brownii*	152677	82094	26526	17531	113	79	30	4	37.0	KY748296
*L. cernuum*	152604	82058	26481	17584	113	79	30	4	37.0	KX354692
*L. davidii* var. *willmottiae*	152659	82060	26498	17603	113	79	30	4	37.0	KX347245
*L. distichum*	152598	82031	26540	17487	113	79	30	4	37.1	KT376489
*L. duchartrei*	152287	81593	26574	17546	113	79	30	4	37.0	KY748300
*L. fargesii*	153235	82217	26990	17038	113	79	30	4	36.9	KX592156
*L. hansonii*	152655	82051	26492	17620	113	79	30	4	37.0	KM103364
*L. henryi*	153119	82480	26553	17533	113	79	30	4	37.0	KY748302
*L. lancifolium*	152574	82007	26492	17583	113	79	30	4	37.0	KY748297
*L. leucanthum*	152935	82476	26550	17361	113	79	30	4	37.0	KY748299
*L. longiflorum*	152793	82230	26520	17523	113	79	30	4	37.0	KC968977
*L. nepalense* var*. ochraceum*	152306	81954	26394	17564	113	79	30	4	37.0	KY748298
*L. sp*.	152715	82065	26516	17618	113	79	30	4	37.0	KM103383
*L. superbum*	152069	81466	26555	17493	113	79	30	4	37.0	KP462883
*L. tsingtauense*	152710	82059	26516	17619	113	79	30	4	37.0	KM103365

### Simple Sequence Repeats (SSR) analysis of the *Lilium* cp genome

We used MISA to detect the SSR sites of all 16 chloroplast genomes. The number of SSRs in chloroplast genomes differed among the sixteen *Lilium* species, as shown in Table 2. The number of SSRs varied from 53 to 78. The most abundant were mononucleotide repeats, which accounted for approximately 56.38% of the total SSRs, followed by dinucleotides and tetranucleotides (Table S2). Hexanucleotides are very rare across the cp genomes.

**Figure 2:**
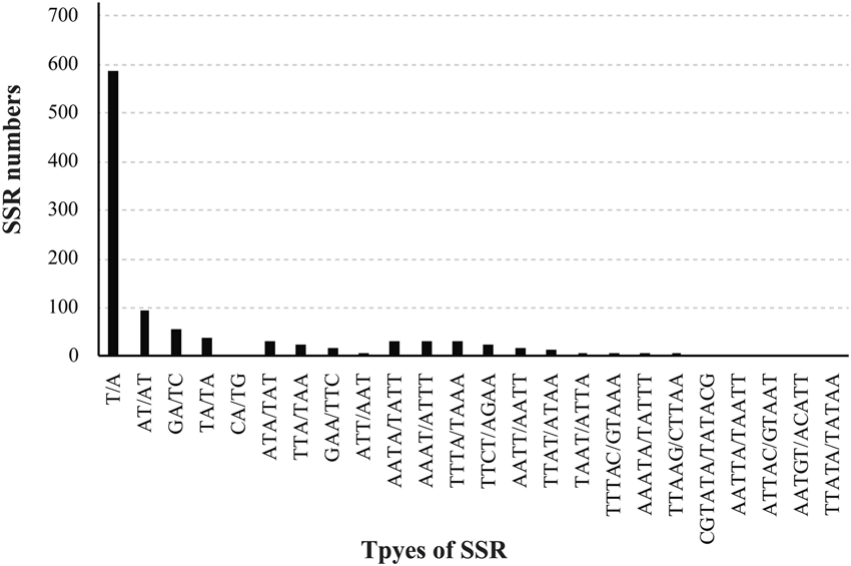
Frequency of simple sequence repeats (SSRs) in the sixteen *Lilium* chloroplast genomes.

In *Lilium*, all mononucleotides (100%) are composed of A/T, and a similar majority of dinucleotides (70.31%) are composed of A/T (Fig. 2). Our findings are comparable to previously reported findings that chloroplast genome SSRs are composed of polyadenine (polyA) or polythymine (polyT) repeats and rarely contained tandem guanine (G) or cytosine (C) repeats. Most of those SSRs are located in the LSC and SSC regions. In general, the SSRs of the sixteen *Lilium* species represent abundant variation and can be used in combination with nuclear SSRs developed in the genus for conservation or reintroduction, species biodiversity assessments and phylogenetic studies of *Lilium* in native or introduced areas.

### Genome sequence divergence among *Lilium* species

We compared nucleotide diversity in the total, LSC, SSC, and IR regions of the cp genomes. The alignment revealed high sequence similarity across the *Lilium* cp genomes, suggesting that they are highly conserved. In total, 3,182 variable sites (2.03%), including 1,449 parsimony-informative sites in the total cp genomes were found (0.93%; Table 3). Among these regions, IR regions exhibite the least nucleotide diversity (0.00093) and SSC higher divergence (0.00839).

**Table 3:** Variable site analyses in *Lilium* chloroplast genomes.

Region	Number of sites	Number of variable sites	Number of parsimony-informative sites	Nucleotide Diversity
LSC	84,935	2281	1066	0.00635
SSC	17,935	665	311	0.00839
IR	26,663	113	42	0.00093
Complete cp genome	156,551	3182	1449	0.00463

The p-distance and number of nucleotide substitutions were used to estimate divergence among the sixteen *Lilium* species. The p-distance among *Lilium* species ranges from 0.0001 to 0.0074, and the number of nucleotide substitutions was found to be 8 to 1,178 (Table S3). *L. fargesii* and *L. longiflorum* show the graest sequence divergence. *L. sp*. (from GenBank) exhibits only 8 nucleotide substitutions (*L. tsingtauense*), with the second lowest divergence being 63 nucleotide substitutions (*L. hansonii*).

### Divergence of hotspot regions

**Figure 3:**
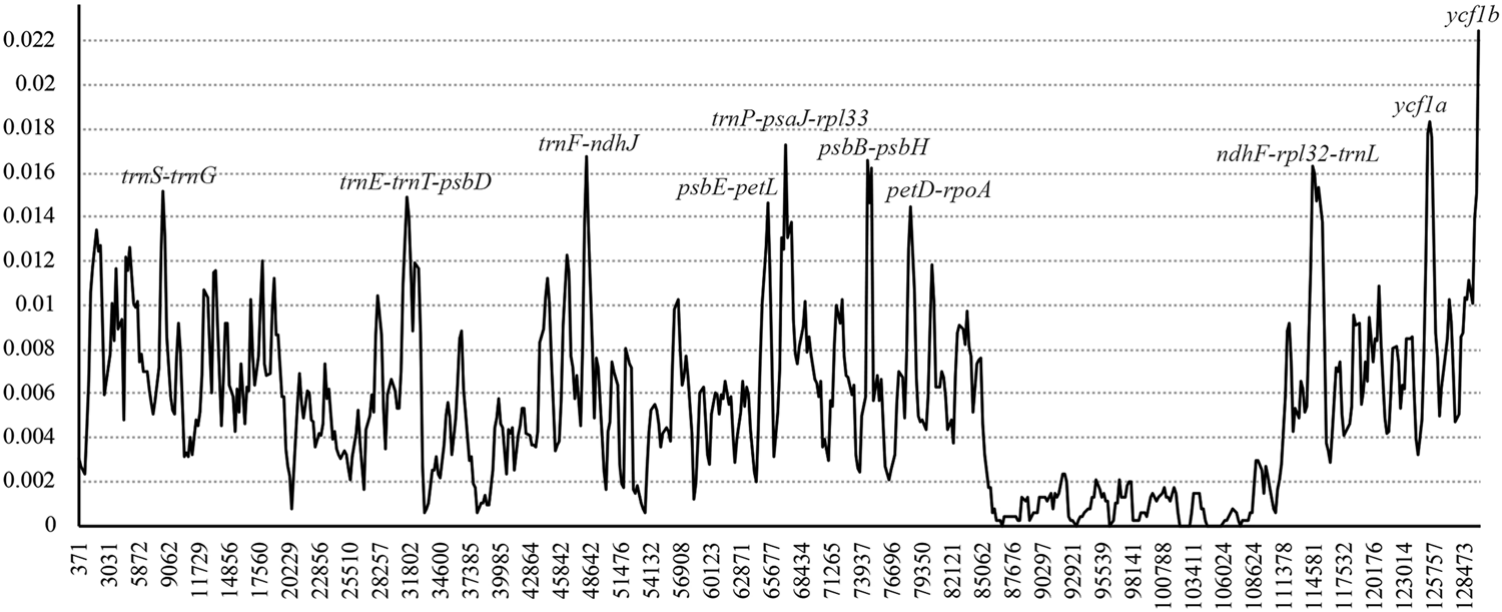
Sliding window analysis of the entire chloroplast genome of 16 *Lilium* species (window length: 600 bp; step size: 200 bp). X-axis: position of the midpoint of a window; Y-axis: nucleotide diversity of each window.

Genome-wide comparative analyses among the sixteen *Lilium* species expected non-coding and SC regions to exhibit higher divergence levels than those of coding and IR regions, respectively (Fig. 3). Furthermore, to calculate the sequence divergence level, the nucleotide diversity (pi) value within 800 bp was calculated (Fig. 3). In the *Lilium* cp genome, these values variy from 0 to 0.02247. We identified 10 hotspot regions for genome divergence that could be utilized as potential markers to reconstruct the phylogeny and plant identification in this genus: *trnS-trnG*, *trnE-trnT-psbD, trnF-ndhJ*, *psbE-petL*, *trnP-psaJ-rpl33*, *psbB-psbH*, *petD-rpoA*, *ndhF-rpl32-trnL*, *ycf1a*, and *ycf1b*. Seven of these (*trnS-trnG*, *trnE-trnT-psbD*, *trnF-ndhJ*, *psbE-petL*, *trnP-psaJ-rpl33*, *psbB-psbH*, and *petD-rpoA*) are located in the LSC, and three (*ndhF-rpl32-trnL*, *ycf1a*, and *ycf1b*) in the SSC region. Only two markers (*ycf1a* and *ycf1b*) are in coding regions. Among these, the coding marker *ycf1b* shows the highest variability (Fig. 3, Table 4).

**Table 4:** Ten regions of highly variable sequences of *Lilium*.

High variable marker	Length	Variable sites	Parsimony informative sites	Nucleotide diversity
*trnS-trnG*	855	55	26	0.01514
*trnE-trnT-psbD*	881	49	21	0.01364
*trnF-ndhJ*	628	35	18	0.01678
*psbE-petL*	755	33	20	0.0146
*trnP-psaJ-rpl33*	665	36	20	0.01726
*psbB-psbH*	1049	49	27	0.01287
*petD-rpoA*	633	35	18	0.0145
*ndhF-rpl32-trnL*	1525	107	50	0.01581
*ycf1a*	1124	70	36	0.0156
*ycf1b*	710	52	23	0.01833
Combine	8825	521	259	0.01533

### Phylogenetic analysis

**Figure 4:**
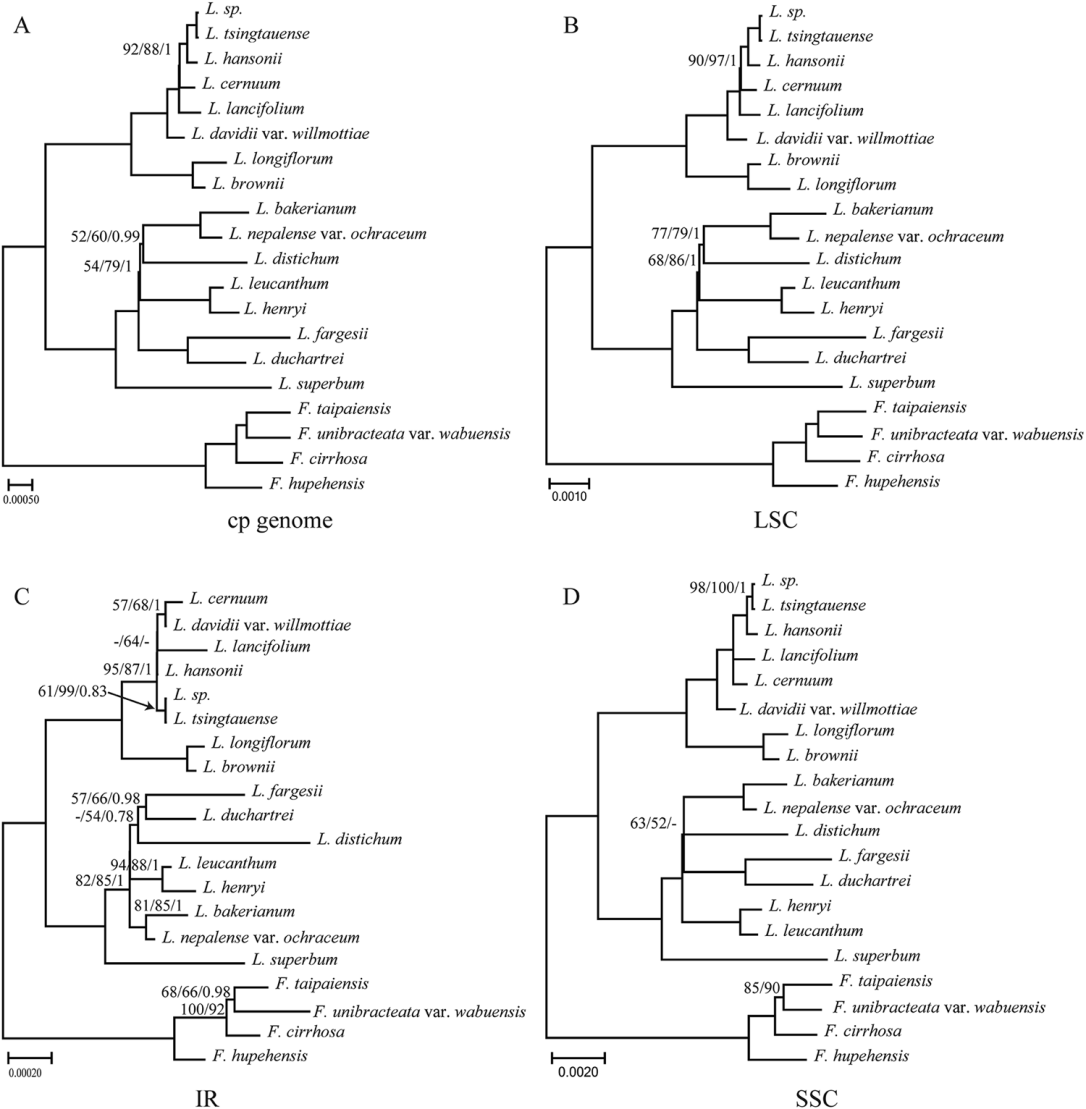
Phylogenetic relationships of the 16 *Lilium* species inferred from maximum parsimony (MP), maximum likelihood (ML) and Bayesian (BI) analyses of different data partitions. (**A**) Whole chloroplast genome. (**B**) LSC region. (**C**) IR region. (**D**) SSC region. Numbers above nodes are support values with MP bootstrap values on the left, ML bootstrap values in the middle, and Bayesian posterior probabilities (PP) values on the right.

**Figure 5:**
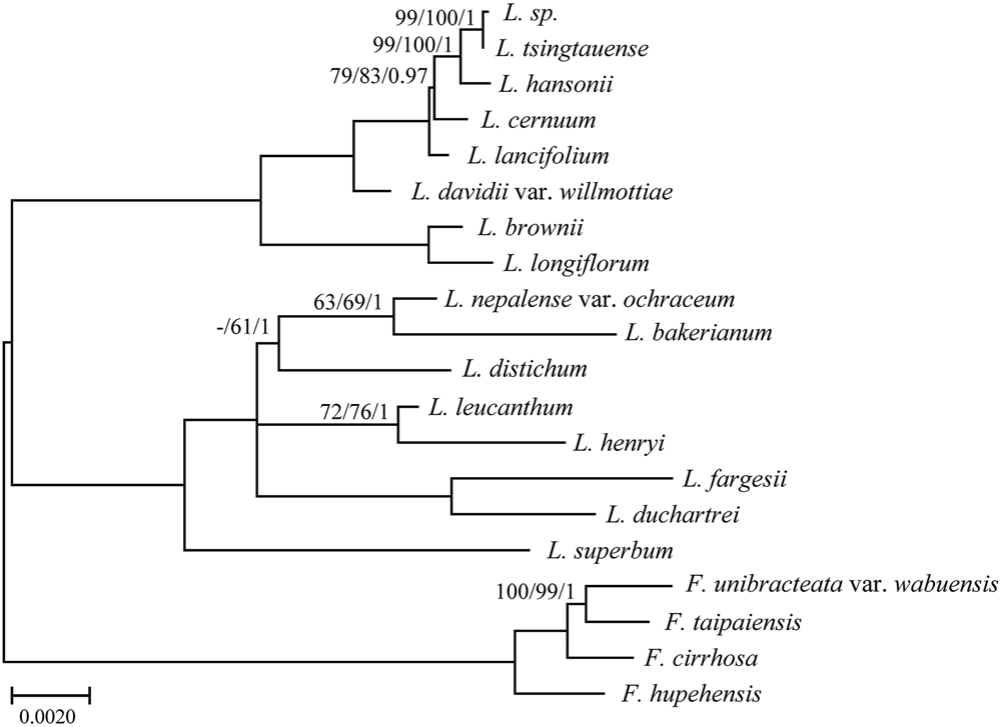
Phylogeny of the 16 *Lilium* species constructed using 10 regions of highly variable sequences. *Fritillaria* was used as the outgroup. Numbers above nodes are support values with MP bootstrap values on the left, ML bootstrap values in the middle, and Bayesian posterior probabilities (PP) values on the right.

In the present study, five datasets (whole complete cp genome sequences, LSC, SSC, IR and ten combined variable regions) from cp genomes of sixteen *Lilium* and four outgroups as well as *Smilax* china were used to perform phylogenetic analysis. Using MP, ML and MrBayes analyses, phylogenetic trees were constructed based on five datasets (Figs 4 and 5, Fig. S1). The topologies based on the three methods of analysis were highly concordant in each dataset, as well as with the results of Rønsted *et al*.^41^ and the phylogenetic trees had moderate to high support, except for the IR dataset, which received poor support. In addition, *Fritillaria* species or added *Smilax china* were used as the outgroup. The results showed that different outgroups could not influence the ingroup topology in our research (Figs 4 and 5, Fig. S1).

The sixteen *Lilium* species were grouped into two branches (Fig. 4). All the datasets indicated that two sect. *Leucolirion* 6b species, *L. brownii* and *L. longiflorum*, form a monophyletic group and then cluster with three sect. *Martagon* species, *L. sp*. (from GenBank), *L. tsingtauense* and *L. hansonii* as well as species of *L. cernuum*, *L. lancifolium* and *L. davidii* var. *willmottiae*, which belong to sect. *Sinomartagon* 6a. In the other branch, *L. superbum* belongs to sect. *Pseudolirium* is distributed in North America; the other seven *Lilium* species are native to Hengduan Mountains and the Himalayas and form another monophyletic clade.

## Discussion

### Chloroplast genome evolution in *Lilium*

In this study, nine new chloroplast genome sequences of *Lilium* were sequenced using the Illumina HiSeq platform. The complete cp genomes range from 151,655 to 153,235 bp, which is within the range of cp genomes from other angiosperms^42^. The cp genomes of *Lilium* are highly conserved, with identical gene content and gene order and genomic structure comprising four parts. Such a low GC content has also been found in other angiosperm chloroplast genomes^43^.

Through a comparative analysis of *Lilium* cp genome sequences, we rapidly developed molecular markers such as single nucleotide polymorphism (SNPs), and SSRs a type of 1–7 nucleotide unit tandem repeat sequence frequently observed in cp genomes, have been shown to have significant potential applications. SSRs are These markers are widely used in population genetics and breeding program studies^44, 45^ because of their high polymorphism even within species, due to slipped-strand mispairing on a single DNA strand during DNA replication^46^. In this study, 1,043 SSRs were identified in sixteen *Lilium* cp genomes. The most abundant are mononucleotide repeats, accounting for more than 56.38% of the total SSRs, followed by the di-, tri-, tetra-, and pentanucleotides. These new resources will be potentially useful for population studies in the *Lilium* genus, possibly in combination with other informative nuclear genome SSRs.

The nucleotide substitution rate is a central question in molecular evolution^47^. Based on the number and distribution of SNP and proportions of variability, the sequence divergence of the IR region is lower than that in LSC and SSC regions, also occurring in many previously reported plants^30, 48^. All pairwise sequence comparisons in our study reveal that DNA sequences evolve at different rates in different species. This result has also been found in other taxa^49^.

Because *Lilium* contains more than 100 species, its DNA barcoding and taxonomy are difficult to assess. The *rbcL*, *matK*, *trnH-psbA*, and *ITS* genes have been widely used to investigate taxonomy and DNA barcoding at the interspecific level (China Plant BOL Group 2014). In DNA barcoding or molecular phylogenetic studies of *Lilium*, these markers had extremely low discriminatory power^11–13^. The indel and SNP mutation events in the genome were not random but clustered as “hotspots.” Such mutational dynamics created the highly variable regions in the genome^30^. Therefore, based on our study, the largest sequence divergence regions are *trnS-trnG*, *trnE-trnT-psbD*, *trnF-ndhJ*, *psbE-petL*, *trnP-psaJ-rpl33*, *psbB-psbH*, *petD-rpoA*, *ndhF-rpl32-trnL*, *ycf1a*, and *ycf1b*. Regions *ycf1a* and *ycf1b* are particularly highly variable among *Lilium*, and they have been added as a core plant DNA barcode^22^. The *trnE-trnT-psbD*, *trnS-trnG* and *ndhF-rpl32-trnL* regions have been widely used for phylogenetic studies^20, 50^. Two rarely reported highly variable regions, *psbB-psbH* and *petD-rpoA*, present in the *Lilium* cp genome were identified in the present study.

### Inferring the phylogeny with chloroplast phylogenomics in *Lilium*

Phylogenetic analyses based on complete plastid genome sequences have provided valuable insights into relationships among and within plant genera. Early studies have been conducted to position uncertain families in angiosperms, such as Amborellaceae^51^, Nymphaeaceae^52^, and Nelumbonaceae^53^. With the recent advent of NGS technology, chloroplast genomes can be sequenced quickly and cheaply, and they have been successfully used to address various phylogenetic questions at the family and even at the species level^54–56^.

In this study, different datasets produced similar topological structures, except the IR dataset, possibly because IR is more conserved and provides fewer information sites than those found in SC regions (Table S2). All trees based on the datasets (except the IR dataset) were not only coincident with the previous phylogenetic studies based on ITS sequences^15, 16^ or the commonly used chloroplast genes such as *matK*, *rbcL*, *atpB* and *atpF*-*H* ^4, 12, 57^ but also had higher bootstrap values and resolution, especially at low classification levels. For example, the *Martagon* clade, including *L. sp*., *L. tsingtauense* and *L. hansonii*, received a higher robust support in the dataset (cp genome, LSC, SSC and ten variable regions) than the clade based on other markers (Figs 4 and 5; Fig. S1). Furthermore, species of *L. sp*. and *L. tsingtauense* were found to form a robustly supported clade ([ML] Bootstrap = 99, [MP] Bootstrap = 100 and PP = 1), suggesting that the two species are likely the same. Phylogenetic trees based on the cp genome, LSC, SSC and ten variable regions datasets support (with low support) that *L. distichum* (from GenBank) form a clade with the clade of *L. bakerianum* and *L. nepalense* var. *ochraceum* or the clade of *L. fargesii* and *L. duchartrei* (Figs 4 and 5; Fig. S1). However, *L. distichum* possesses a whorled leaf and is attributed to the sect. *Martagon*. Therefore, the species identification of *L. distichum* from GenBank may be inaccurate. However, evolutionary relationships and divisions within species/section need further investigation.

This study used the cp genome data to infer the phylogenetic relationships in *Lilium*, providing genome-scale support. The cp genome is expected to be useful in resolving the deeper branches of the phylogeny as more whole-genome sequences become available in *Lilium*.

## Methods

### Plant material and DNA extraction

Fresh leaves of nine *Lilium* species were sampled (Table 5). Specimens were deposited in the herbarium of the Institute of Botany, Chinese Academy of Sciences (PE) (Table 5). Total genomic DNA was extracted using a plant genome extraction kit (Tiangen, Beijing, China). Subsequently, DNA concentration was measured using a NanoDrop spectrophotometer 2000 (Thermo Fisher Scientific, America).

**Table 5:** Sampled species and their voucher specimens used in this study.

Species	Section	Voucher	Locality
*L. fargesii*	Lophophorum	BOP040593	Shaanxi
*L. brownii*	Leucolirion 6b	BOP040602	Hubei
*L. lancifolium*	Sinomartagon	BOP040607	Hubei
*L. nepalense* var*.ochraceum*	Lilium–Nomocharis	BOP040618	Yunnan
*L. leucanthum*	Leucolirion 6a	BOP040622	Chongqing
*L. davidii* var*. willmottiae*	Sinomartagon	BOP040624	Yunan
*L. duchartrei*	Sinomartagon	BOP040925	Sichuan
*L. bakerianum*	Lophophorum	BOP040929	Yunnan
*L. henryi*	Leucolirion 6a	BOP040933	Hubei

### Genome sequencing, assembly and annotation

DNA was sheared to construct a 400 bp (insert size) paired-end library in accordance with the Illumina HiSeq 4000 standard protocol. The paired-end reads were qualitatively assessed and assembled using SPAdes 3.6.1^58^. The gaps were filled by PCR amplification and Sanger sequencing. Sanger sequence reads were proofread and assembled with Sequencher 4.10 (http://www.genecodes.com).

All genes encoding proteins, transfer RNAs (tRNAs), and ribosomal RNAs (rRNAs) were annotated on *Lilium*. Plastomes were annotated using Dual Organellar Genome Annotator (DOGMA) software and the tRNAscan-SE 1.21 program^59, 60^. Initial annotation, putative starts, stops, and intron positions were determined by comparison with homologous genes in other *Lilium* cp genomes.

### Microsatellite analysis

Perl script MISA^61^ was used to detect microsatellites (mono-, di-, tri-, tetra-, penta-, and hexanucleotide repeats) with the following thresholds (unit size, min repeats): ten repeat units for mononucleotide SSRs, five repeat units for dinucleotide SSRs, four repeat units for trinucleotide SSRs, and three repeat units each for tetra-, penta-, and hexanucleotide SSRs.

### Molecular marker identification and sequence divergence analysis

The sequences were first aligned using MAFFT v7^62^ and then manually adjusted using BioEdit software. Subsequently, a sliding window analysis was conducted to evaluate the nucleotide variability (Pi) of the cp genome using DnaSP version 5.1 software^63^. The step size was set to 200 base pairs, and the window length was set to 600 base pairs.

Variable and parsimony-informative base sites across the complete cp genomes and the large single copy (LSC), small single copy (SSC), and inverted repeat (IR) regions of the six cp genomes were calculated using MEGA 6.0 software^64^. The p-distance among *Lilium* cp genomes was calculated to evaluate the divergence of *Lilium* species using MEGA software.

### Phylogenetic analysis

Phylogenetic trees were constructed by maximum parsimony (MP), maximum likelihood (ML) and Bayesian analysis (BI) methods using the entire cp genome, LSC, SSC, IR regions and combining ten variable regions. The lengths of all alignment matrices of these datasets are shown in Table 3. In all phylogenetic analyses, *Fritillaria* or *Smilax china* were used as the outgroup (Figs 4 and 5, Fig. S1).

MP analyses were conducted using PAUP v4b10^65^ with heuristic searches with the ‘MulTrees’ option followed by tree bisection–reconnection (TBR) branch swapping. Branch support was assessed with 1,000 random addition replicates. All characters were unordered and were accorded equal weight, with gaps being treated as missing data. The best-fit substitution models were selected by running ModelTest 3.7^66^ under the Akaike information criterion (AIC). ML analyses were performed using RAxML-HPC BlackBox v.8.1.24 at the CIPRES Science Gateway website^67, 68^. For ML analyses, the best-fit models, general time reversible (GTR) + G, were used in all analyses, as suggested with 1,000 bootstrap replicates. BI was performed with MrBayes 3.2^69^. Two independent Markov chain Monte Carlo (MCMC) chains were run, each with three heated and one cold chain for 50 million generations. Each chain started with a random tree, default priors and sampling trees every 1,000 generations, with the first 25% discarded as burn-in. Stationarity was considered reached when the average standard deviation of split frequencies remained below 0.01.

## Electronic supplementary material

Supplementary Information
