# Supplementary material for: Complete chloroplast genome sequences of Lilium: insights into evolutionary dynamics and phylogenetic analyses
Source: Sci Rep. 2017 Jul 18;7:5751. doi: 10.1038/s41598-017-06210-2 (PMC5515919; doi:10.1038/s41598-017-06210-2)

Complete chloroplast genome sequences of *Lilium*: insights into evolutionary dynamics and phylogenetic analyses

Yun-peng Du^1^, Yu Bi^1,2^, Feng-ping Yang^1^, Ming-fang Zhang^1^, Xu-qing Chen^1^, Jing Xue^1^, Xiu-hai Zhang^1^*

1. Beijing Agro-Biotechnology Research Center, Beijing Key Laboratory of Agricultural Genetic Resources and Biotechnology; Key Laboratory of Urban Agriculture (North), Ministry of Agriculture, Beijing Academy of Agriculture and Forestry Sciences, Beijing 100097, China

2. School of Horticulture, Jilin Agricultural University, Changchun, Jilin Province

130000, China

*Corresponding author. E-mail: zhangxiuhai@baafs.net.cn

[Tel: 86-10-51503829](tel:86-10-51503829)

Fax: 86-10-51503980

**Supplementary information**: Table S1. List of genes in the chloroplast genome of *Lilium*.

| Category for genes | Group of gene | Name of gene |
| --- | --- | --- |
| Photosynthesis related genes | Photosystem Ⅰ | *psaA, psaB, psaC, psaI, psaJ* |
|  | Photosystem Ⅱ | *psbA, psbB, psbC, psbD, psbE, psbF, psbH, psbI, psbJ, psbK, psbL, psbN, psbT, psbZ* |
|  | cytochrome b/f compelx | *petA, *petB, *petD, petG, petL, petN* |
|  | ATP synthase | *atpA, atpB, atpE, *atpF, atpH, atpI* |
|  | cytochrome c synthesis | *ccsA* |
|  | Assembly/stability of photosystem Ⅰ | **ycf3,ycf4* |
|  | NADPH dehydrogenase | **ndhA, *ndhB, ndhC, ndhD, ndhE, ndhF ,ndhG, ndhH, ndhI, ndhJ, ndhK* |
|  | Rubisco | *rbcL* |
| Transcription and translation related genes | transcription | *rpoA, rpoB, *rpoC1, rpoC2* |
|  | ribosomal proteins | *rps2, rps3, rps4, rps7, rps8, rps11, *rps12, rps14,rps15, *rps16, rps18, rps19, *rpl2, rpl14, *rpl16, rpl20, rpl22, rpl23, rpl32, rpl33,rpl36* |
| RNA genes | ribosomal RNA | *rrn5, rrn4.5, rrn16, rrn23* |
|  | transfer RNA | **trnA-UGC, trnC-GCA, trnD-GUC, trnE-UUC, trnF-GAA,trnG-GCC, *trnG-UCC, trnH-GUG, trnI-CAU, *trnI-GAU,*trnK-UUU, trnL-CAA, *trnL-UAA, trnL-UAG, trnfM-CAU,trnM-CAU, trnN-GUU, trnP-UGG, trnQ-UUG,trnR-ACG, trnR-UCU, trnS-GCU, trnS-GGA, trnS-UGA, trnT-GGU,trnT-UGU, trnV-GAC, *trnV-UAC, trnW-CCA, trnY-GUA* |
| Other genes | RNA processing | *matK* |
|  | carbon metabolism | *cemA* |
|  | fatty acid synthesis | *accD* |
|  | proteolysis | **clpP* |
| Genes of unknown function | conserved reading frame | *ycf1, ycf2* |

Table S2. Types and numbers of SSRs in *Lilium* chloroplast genomes.

| Species | mononucleotide | dinucleotide | trinucleotide | tetranucleotide | pentanucleotide | hexanucleotide | Total |
| --- | --- | --- | --- | --- | --- | --- | --- |
| *L. bakerianum* | 35 | 10 | 5 | 12 | 0 | 0 | 62 |
| *L. brownii* | 40 | 12 | 5 | 9 | 0 | 0 | 66 |
| *L. cernuum* | 39 | 14 | 5 | 8 | 2 | 0 | 68 |
| *L. davidii* var*. willmottiae* | 41 | 13 | 6 | 7 | 1 | 0 | 68 |
| *L. distichum* | 25 | 11 | 5 | 11 | 2 | 0 | 54 |
| *L. duchartrei* | 32 | 10 | 4 | 11 | 3 | 0 | 60 |
| *L. fargesii* | 50 | 11 | 4 | 11 | 2 | 0 | 78 |
| *L. hansonii* | 34 | 14 | 6 | 8 | 2 | 0 | 64 |
| *L. henryi* | 39 | 10 | 6 | 12 | 1 | 0 | 68 |
| *L. lancifolium* | 37 | 13 | 5 | 8 | 1 | 0 | 64 |
| *L. leucanthum* | 41 | 11 | 6 | 12 | 2 | 0 | 72 |
| *L. longiflorum* | 34 | 15 | 5 | 9 | 1 | 0 | 64 |
| *L. nepalense* var*. ochraceum* | 46 | 10 | 5 | 12 | 1 | 0 | 74 |
| *L. sp.* | 34 | 13 | 6 | 8 | 2 | 1 | 64 |
| *L. superbum* | 27 | 12 | 4 | 9 | 1 | 0 | 53 |
| *L. tsingtauense* | 34 | 13 | 6 | 8 | 2 | 1 | 64 |
|  | 588 | 192 | 83 | 155 | 23 | 2 | 1043 |

Table S3. Numbers of nucleotide substitutions and sequence distance in 16 complete cp genomes. The upper triangle indicates the number of nucleotide substitutions, and the lower triangle indicates the number of sequence distances in complete cp genomes.

|  | *lancifolium* | *cernuum* | *davidii* var*. willmottiae* | *tsingtauense* | *sp.* | *hansonii* | *brownii* | *longiflorum* | *superbum* | *nepalense* var*. ochraceum* | *leucanthum* | *henryi* | *fargesii* | *duchartrei* | *bakerianum* | *distichum* |
| --- | --- | --- | --- | --- | --- | --- | --- | --- | --- | --- | --- | --- | --- | --- | --- | --- |
| *lancifolium* |  | 115 | 154 | 125 | 125 | 120 | 426 | 489 | 1103 | 935 | 940 | 978 | 1130 | 988 | 992 | 1019 |
| *cernuum* | 0.0007 |  | 131 | 102 | 102 | 97 | 406 | 474 | 1089 | 992 | 930 | 960 | 1117 | 974 | 975 | 1004 |
| *davidii* var*. willmottiae* | 0.0009 | 0.0008 |  | 147 | 147 | 142 | 375 | 439 | 1061 | 895 | 899 | 930 | 1079 | 940 | 946 | 973 |
| *tsingtauense* | 0.0008 | 0.0007 | 0.0009 |  | 8 | 63 | 412 | 478 | 1101 | 930 | 939 | 976 | 1129 | 988 | 986 | 1020 |
| *sp.* | 0.0008 | 0.0007 | 0.0009 | 0.0001 |  | 63 | 412 | 478 | 1102 | 932 | 941 | 978 | 1129 | 990 | 988 | 1022 |
| *hansonii* | 0.0008 | 0.0006 | 0.0008 | 0.0004 | 0.0004 |  | 408 | 469 | 1097 | 929 | 934 | 975 | 1128 | 982 | 979 | 1019 |
| *brownii* | 0.0026 | 0.0025 | 0.0023 | 0.0025 | 0.0025 | 0.0025 |  | 138 | 1114 | 957 | 956 | 996 | 1142 | 994 | 1004 | 1038 |
| *longiflorum* | 0.0030 | 0.0029 | 0.0027 | 0.0029 | 0.0029 | 0.0029 | 0.0009 |  | 1155 | 978 | 966 | 1006 | 1178 | 1023 | 1040 | 1057 |
| *superbum* | 0.0071 | 0.0070 | 0.0069 | 0.0071 | 0.0071 | 0.0071 | 0.0072 | 0.0074 |  | 761 | 765 | 814 | 962 | 831 | 833 | 856 |
| *nepalense* var*. ochraceum* | 0.0060 | 0.0059 | 0.0057 | 0.0059 | 0.0059 | 0.0059 | 0.0060 | 0.0062 | 0.0049 |  | 473 | 517 | 675 | 539 | 205 | 548 |
| *leucanthum* | 0.0059 | 0.0058 | 0.0056 | 0.0059 | 0.0059 | 0.0058 | 0.0060 | 0.0060 | 0.0049 | 0.0028 |  | 123 | 690 | 549 | 552 | 548 |
| *henryi* | 0.0062 | 0.0061 | 0.0059 | 0.0062 | 0.0062 | 0.0061 | 0.0062 | 0.0063 | 0.0052 | 0.0031 | 0.0007 |  | 691 | 561 | 544 | 597 |
| *fargesii* | 0.0072 | 0.0071 | 0.0069 | 0.0072 | 0.0072 | 0.0072 | 0.0072 | 0.0074 | 0.0062 | 0.0043 | 0.0043 | 0.0042 |  | 472 | 705 | 770 |
| *duchartrei* | 0.0063 | 0.0062 | 0.0060 | 0.0063 | 0.0063 | 0.0063 | 0.0064 | 0.0065 | 0.0054 | 0.0034 | 0.0034 | 0.0033 | 0.0030 |  | 561 | 629 |
| *bakerianum* | 0.0062 | 0.0061 | 0.0059 | 0.0062 | 0.0062 | 0.0062 | 0.0063 | 0.0065 | 0.0053 | 0.0013 | 0.0034 | 0.0033 | 0.0045 | 0.0036 |  | 610 |
| *distichum* | 0.0065 | 0.0064 | 0.0062 | 0.0065 | 0.0065 | 0.0065 | 0.0066 | 0.0067 | 0.0055 | 0.0034 | 0.0034 | 0.0037 | 0.0049 | 0.0040 | 0.0039 |  |

Figure S1. Phylogeny of the 16 *Lilium* species constructed using 10 regions of highly variable sequences. *Fritillaria* and *Smilax china* were used as the outgroup. Numbers above nodes are support values with MP bootstrap values on the left, ML bootstrap values in the middle, and Bayesian posterior probabilities (PP) values on the right.


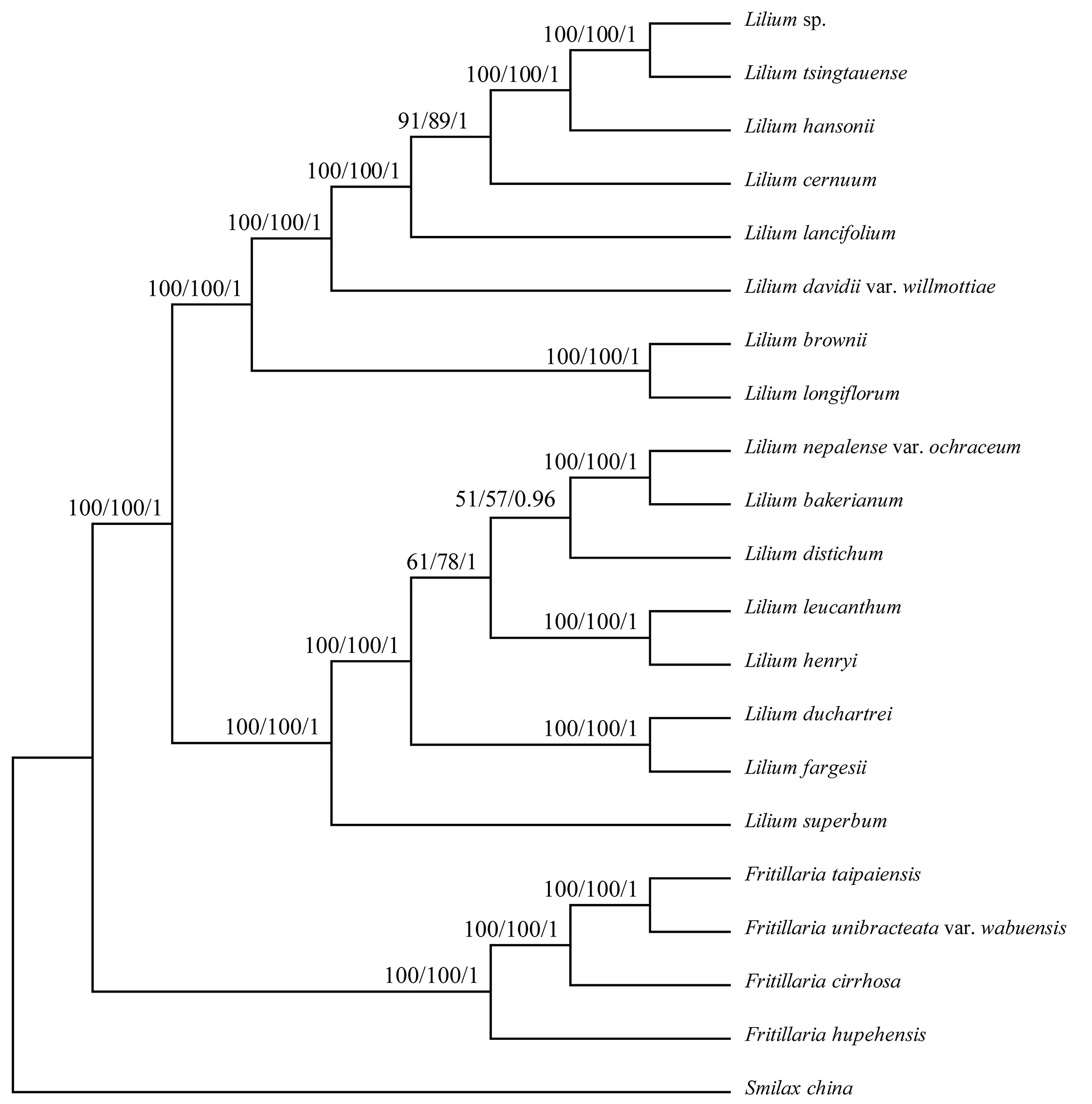

Supplement: Supplementary file 1 — Supplementary Information [file 41598_2017_6210_MOESM1_ESM.doc]
